# The varieties of delusional syndrome of possession in schizophrenia

**DOI:** 10.1192/j.eurpsy.2021.2040

**Published:** 2021-08-13

**Authors:** O. Borisova, G. Kopeyko, E. Gedevani, I. Samsonov, V. Kaleda

**Affiliations:** 1 Investigation Group Of Specific Psychopathological Forms At Department Of Youth Psychiatry, Federal State Budgetary Scientific Institution «Mental Health Research Center», Moscow, Russian Federation; 2 Researching Group Of Specific Forms Of Mental Disorders, FSBSI Mental Health Research Center, Moscow, Russian Federation; 3 Department Of Youth Psychiatry, FSBSI «Mental Health Research Centre», Moscow, Russian Federation

**Keywords:** schizophrénia, possession, religious delusions, psychopathology

## Abstract

**Introduction:**

Delusional Syndrome of Possession in schizophrenia (DSPS) is insufficiently explored. Although it characterized by significant severity of clinical state and resistance to psychopharmacotherapy, and may be accompanied by high social risks.

**Objectives:**

To carry out clinical and psychopathological differentiation of DSPS and to define its personalized diagnostic and prognostic criteria.

**Methods:**

66 patients with DSPS were observed (F20.0, F20.01, F20.02 according to ICD-10) by psychopathological, psychometrical and statistical methods.

**Results:**

Persistent delusional conviction of patient in invasion of certain «spiritual being» (demonic or divine) inside of the body and soul is the specific core of DSPS. The psychotic episode with DSPS has similar pattern with paranoid syndrome of Kandinsky–Clérambault. Although, the structure of the syndrome is varying, and characterized by predominance of hallucinatory or delusion symptoms. According to these varieties two different types of DSPS were identified, which were observed in continuous or paroxysmal course of disease. The forms of destructive delusional behavior were also different for both of these types.

**Conclusions:**

Delusional Syndrome of Possession in schizophrenia (DSPS) is complex and diverse phenomenon, due to religious content of delusional disorders, which occurs in specific psychopathological structure of psychotic state. This fact may cause controversy both in psychiatric practice and in religious communities. So, the obtained data could be important for social and treatment predicting, as well as for pastoral counseling.

**Disclosure:**

No significant relationships.

